# Predicting gut microbiota dynamics in obese individuals from cross-sectional data

**DOI:** 10.3389/fcimb.2025.1485791

**Published:** 2025-06-10

**Authors:** Ena Melvan, Andrew P. Allen, Tea Vuckovic, Irena Soljic, Antonio Starcevic

**Affiliations:** ^1^ School of Natural Sciences, Macquarie University, Sydney, NSW, Australia; ^2^ Research and Development Department, Metabelly, Split, Croatia; ^3^ Faculty of Food Technology and Biotechnology, University of Zagreb, Zagreb, Croatia

**Keywords:** gut microbiota, obesity, microbial interactions, personalized nutrition, GLV method, dietary interventions, microbiome dynamics

## Abstract

**Introduction:**

Obesity affects approximately 39% of adults worldwide. While gut microbiota has been linked to obesity, most research has focused on static taxonomic composition rather than the dynamic interactions between microbial taxa.

**Methods:**

We applied BEEM-Static, a generalized Lotka-Volterra model, to cross-sectional 16S rRNA gut microbiome data from six public datasets, comprising 2,435 profiles from lean and obese individuals.

**Results:**

A total of 57 significant microbial interactions were identified in obese individuals (79% negative), compared to 37 in lean individuals (92% negative). For example, Bacteroidetes showed a stronger inhibitory effect on Firmicutes in obese individuals (−0.41) than in lean ones (−0.26). Firmicutes and Proteobacteria exhibited consistently higher carrying capacities in obese populations.

**Discussion:**

These findings suggest that microbial interaction networks—not just taxonomic abundance—play a key role in obesity-related dysbiosis. Our approach enables the inference of microbiota dynamics from a single time point, paving the way for tailored dietary interventions, which we refer to as *Optibiomics*.

## 1 Introduction

Obesity currently affects nearly 40% of adults worldwide, totaling around 1.9 billion people (World Health Organization, 2015). It represents a global public health challenge because it is known to adversely affect mental health and to increase the risk of death due to related diseases including diabetes, cardiac disease, stroke, and some forms of cancer (Jung, 1997; Stein and Colditz, 2004; James et al., 2001). Growing evidence suggests that gut microbiota significantly contributes to the negative health impacts of obesity through various mechanisms, including neurotransmitter production, digestion, and the regulation of satiety (Walters et al., 2014; Maruvada et al., 2017; John and Mullin, 2016; Tseng and Wu, 2019; Castaner et al., 2018; Sekirov et al., 2010). Alterations in gut microbiota composition, which are influenced by dietary factors, play a very important role in the development of obesity and its associated ailments (Alou et al., 2016).

Currently, our understanding of the gut microbiome’s function in health and disease is based primarily on correlations between disease status and the taxonomic composition of microbiota. Despite extensive research, other role of microbial interactions in human diseases remains limited. Relatively little is known about the role of interactions among microbes in human diseases (Chen et al., 2020). Given the wide range of host–microbe interactions linked to health, dysbiosis, polymicrobial infections, and single-agent infections, understanding interactions among microbes is critical to predicting dynamics of the microbiota. Developing such a mechanistic understanding and how it relates to human health promises to aid in the development of therapeutic methods to prevent dysbiosis and obesity, ultimately enabling the selection of precise dietary interventions to promote beneficial shifts in the gut microbiota.

It has been suggested that changes in the relative abundances of *Actinobacteria*, *Bacteroidetes*, *Firmicutes*, and *Proteobacteria* occur in response to obesity (Walters et al., 2014; Maruvada et al., 2017; John and Mullin, 2016; Tseng and Wu, 2019; Castaner et al., 2018). Although the taxonomic names *Bacteroidetes* and *Firmicutes* were changed recently to *Bacteroidota* and *Firmicutes* A & B, respectively among others, in this manuscript we have preferred to use the original names for the sake of consistency and clarity (Oren and Garrity, 2021). *Actinobacteria* has been found to play an important role in the pathophysiology of metabolic disorders, most notably obesity, so we would expect relative abundance for this group to be higher in obese people (Tseng and Wu, 2019; Castaner et al., 2018). Recent research suggests that *Actinobacteria* can have both positive and negative effects on health, depending on the host’s metabolic condition (Tseng and Wu, 2019; Castaner et al., 2018). These bacteria can enhance gut health in lean individuals through fermentation processes, while in obese individuals, certain strains may lead to inflammation and dysbiosis. The *Firmicutes*-to-*Bacteroidetes* abundance ratio is frequently mentioned as a biomarker that tends to be higher in obese individuals (John and Mullin, 2016; Tseng and Wu, 2019; Castaner et al., 2018). Inflammation in the large bowel occurs in response to dysbiosis, which is often characterized by an overgrowth of facultative anaerobic *Proteobacteria* (Winter and Bäumler, 2014), thus the relative abundance of *Proteobacteria* is also expected to increase with obesity (Walters et al., 2014; Maruvada et al., 2017; John and Mullin, 2016; Tseng and Wu, 2019; Castaner et al., 2018). In summary, these findings suggest a decline in the abundance of *Bacteroidetes* relative to other phyla in response to obesity. Given that dietary patterns have been shown to significantly impact the distribution of these bacteria, understanding how various diets affect microbial dynamics is crucial for developing tailored nutritional approaches aimed at restoring the normal balance of microorganisms within the gut (Zeevi et al., 2015). However, these expectations are challenged by a 2014 meta-analysis (Walters et al., 2014), which found no consistent relationships between obesity and microbiome composition across five databases analyzed using uniform methods. Their conclusions highlighted the need to consider alternative statistical approaches of assessing relationships of microbiota to obesity that go beyond assessing differences in taxonomic composition using traditional statistical approaches.

Alternative statistical approaches that are motivated by dynamic mathematical models afford new opportunities to infer processes from patterns in microbiota data (Sender et al., 2016) and may yield insights into how the microbiome affects human health and disease (Kumar et al., 2019). Dynamical models are typically parameterized using longitudinal data that are collected using experiments that assay microbiota in one or more subjects at multiple time points (Maruvada et al., 2017). These experiments differ from cross-sectional research studies, which involve collecting data from many subjects at a single point in time to seek general patterns while maintaining a reasonable false discovery rate (Maruvada et al., 2017). Given the relative scarcity of longitudinal studies and data, using ecological models based on generalized Lotka-Volterra models (GLVMs), such as BEEM-Static, allow for a dynamic insight to be obtained from cross-sectional data (Metz et al., 1995; Hofbauer and Sigmund, 1998; Li et al., 2021) [16-18]. This capability is particularly crucial given the challenges in collecting longitudinal data, as it allows for meaningful insights and predictions to be derived from a single time point, making the approach more practical and scalable in clinical and research settings.

BEEM-Static was used to extract directed bacterial interactions from cross-sectional microbiome profiling data (Li et al., 2021). BEEM-Static is an R package that uses the generalized Lotka-Volterra (GLVM) model to infer microbial interactions from cross-sectional microbiome profiling data. The GLVM equations are first-order nonlinear differential equations that predict changes in population abundance through time based on fitted parameters for growth rates, carrying capacities, and interspecific interactions among taxa comprising the microbial community (Li et al., 2016; Kumar et al., 2019). BEEM-Static is an extension of the recently proposed BEEM algorithm, which works with longitudinal microbiome sequencing data (Li et al., 2019). Of particular relevance is that even though the GLVM parameters apply to the dynamics of absolute abundance, BEEM-Static facilitates estimation of the GLVM parameters from relative (i.e. proportional) abundance data, making it suitable for microbiome datasets. This approach is particularly valuable in understanding how specific dietary components modulate the gut microbiota’s structure and function, offering insights that could lead to the development of AI-driven dietary recommendations tailored to individual microbiome profiles (David et al., 2014). BEEM-Static was chosen for this analysis due to its ability to infer ecological interactions from cross-sectional data, which are more readily available and easier to obtain than longitudinal datasets. While this approach offers a practical and scalable method for analyzing microbiome dynamics, it does not capture temporal changes, highlighting the need for complementary longitudinal studies to fully understand microbiome behavior over time.

For this study, we separately applied the BEEM-Static algorithm to microbiome data collected from lean and obese individuals and then assessed whether the inferred carrying capacities and inter-specific interactions exhibited significant differences. Interactions among organisms are varied (Malcolm, 1966), essential to the maintenance of community structure, and can be classified as being either positive or negative depending on whether a numerical increase in the abundance of one species population results in an increase or decrease in a co-occurring species (Jones et al., 1997). Analysis revealed that Actinobacteria exhibited varying interaction patterns between lean and obese populations (Walters et al., 2014). Specifically, while some strains were positively correlated with beneficial metabolic functions in lean individuals, they were associated with negative interactions in obese individuals, potentially contributing to metabolic disorders (Tseng and Wu, 2019). We hypothesize that established differences in the intestinal environments of lean versus obese people (e.g. availability of substrates, pH, reduction potential) will affect the carrying capacities of particular taxa and the interactions among taxa.

## 2 Material and methods

### 2.1 Datasets

This analysis was conducted using 6 publicly available human gut microbiome datasets, each of which contains counts of operational taxonomic units (OTUs) derived from 16s rRNA gene sequences for both lean and obese people (**Table 1**). Samples were analyzed from a total of 1148 lean individuals and 1287 obese individuals. The selection of these datasets was based on their comprehensive representation of gut microbial profiles across different populations and the presence of lean and obese individuals in each, making it possible to carry out meaningful cross-comparisons. These datasets were obtained utilizing a variety of sequencing systems, including Illumina and 454 pyrosequencing. To reduce platform-specific technical heterogeneity, we aggregated data at the phylum level and used the same preprocessing strategy (Walters et al., 2014) for all datasets. This degree of taxonomic precision has been shown to be robust in cross-platform comparisons (Walters et al., 2014). Inclusion requirements also required the availability of BMI metadata, a sufficient sample size, and public accessibility. The six datasets provide a broad overview of the gut microbiome in lean, overweight, and obese individuals, encompassing diverse age ranges, phenotypic categories, and some sex distribution data. Out of the six datasets, four (American Gut, Ross, HMP, and Goodrich) provided gender distribution data, revealing a total of 4,165 female and 3,899 male participants across all groups. The remaining two datasets (Turnbaugh and Gordon) did not include gender distribution, representing 7.0% of the total samples. In terms of phenotypes, the datasets include a total of 5,157 samples classified as H (Healthy) and 5,909 samples classified as OB (Obese), summing lean and overweight individuals into the obese category. Age data were available for most datasets, covering a broad range from 0 to 93 years, with mean ages varying between datasets: 25.9 to 61.8 years for healthy individuals and 28.0 to 61.3 years for obese individuals. Obesity is defined as abnormal or excessive fat accumulation that presents a risk to health. An individual is generally considered overweight if they have a body mass index (BMI) value exceeding 25 kg m^-2^ and obese if they have a value exceeding 30 kg m^-2^. Due to data limitations, for the purposes of this study, we classified an individual as “lean” if they were identified as such or had a reported BMI less than or equal to 25 kg m^-2^, and “obese” if they were reported as being overweight or obese or had a reporting BMI exceeding 25 kg m^-2^. However, it also proves that diet affects the microbiota and, thus, while this analysis is confined to the evaluation of microbiota composition and interactions, the differences in dietary habits among the populations studied might influence the patterns of microbial dynamics. While we aimed to control for potential confounding factors such as dietary habits and geographic differences, we recognize that these factors may still influence our findings. Future research should strive for more balanced sample sizes and include detailed dietary assessments. Dietary components such as fiber, lipids, and proteins have been proven to have a significant effect on gut microbiota composition. High-fiber diets promote the growth of beneficial bacteria such as *Bacteroidetes* and *Firmicutes*, which have been associated with improved metabolic health (Zeevi et al., 2015). Saturated fat-rich meals, on the other hand, may promote pathogenic bacteria, contributing to dysbiosis and obesity (Castaner et al., 2018). Clinical studies have demonstrated that dietary interventions can lead to measurable changes in microbiota composition, reinforcing the notion that diet is a critical factor in shaping microbial dynamics (Walters et al., 2014; Tseng and Wu, 2019).

**Table 1:** Numbers of samples and taxa included in the present meta-analysis.

Dataset	Sample size			OTUs prior to data exclusion	Phyla analyzed	Phyla excluded due to low prevalence
	Total	Lean	Obese			
Gordon (Turnbaugh et al., 2009)	281	61	220	219	3	3
Goodrich (Goodrich et al., 2014)	1017	489	528	877	7	8
Ross (Ross et al., 2015)	63	26	37	211	5	5
Turnbaugh (Turnbaugh et al., 2009)	142	35	107	687	4	4
AG (McDonald et al., 2018)	711	320	210	443	4	5
HMP (Turnbaugh et al., 2007)	402	217	185	166	4	4

The pre-processed datasets and codes can be found in https://github.com/enmelvan/Chapter4.

Counts in each database were aggregated from the OTU level to the phylum level in R Studio Version 1.4.1717 using the R package phyloseq (McMurdie and Holmes, 2013). The model was built at all taxonomic levels for each individual database, with the phylum level showing the highest coefficient of determination (R²). Given this, the phylum level was chosen as the baseline for analysis, offering a balance between identifying microbial interactions and maintaining model performance and interpretability. Taxa within phyla appear to show differential responses to obesity status, but we focused on the phylum level to facilitate comparisons. Phyla were only included in the analysis if they were found in at least 33% of the samples for that database (**Table 1**), which is somewhat more stringent than the often-used criterion of 25% prevalence (Li et al., 2021). Using the 25% criterion would have resulted in the inclusion of 1 additional phylum (*Verrucomicrobia*) in the analysis of HMP dataset, and of 1 additional phylum (*Proteobacteria*) in the analysis of the Gordon dataset. Because we had a limited number of samples and BEEM-static had been tested on over 4,000 microbiome data points, we decided to use 37% prevalence as a criterion. The same OTUs are still significant at 25% and 33% prevalence, and because they are the most abundant overall, any pruning variant in between these criteria would result in the same outcome. BEEM-static detected microbiomes that were not in equilibrium and automatically deleted them from further investigation.

### 2.2 Statistical analysis

BEEM-Static takes as input an OTU table of counts comprised of samples (in columns) and taxa (in rows). While BEEM-Static can provide useful insights by inferring ecological interactions from cross-sectional data, it is essential to acknowledge its limitations. The gut ecosystem is extraordinarily dynamic and responds to various environmental pressures, making it susceptible to complex microbial interactions that can change over time. Although our goal was to identify ‘key players’ that distinguish between obese and lean populations, we recognize that BEEM-Static cannot fully capture the temporal dynamics inherent in microbiome changes, particularly in response to dietary interventions aimed at preventing or treating obesity. Longitudinal studies using dynamical models are better suited for understanding these complexities because they can track changes over time and provide insights into how interventions impact microbiome dynamics. For instance, our previous work demonstrated the importance of longitudinal analysis in understanding urinary microbiota dynamics (Ceprnja et al., 2021). However, such models require substantial collection of data and may be more susceptible to individual response variability. As a result, while our approach offers a practical method of analyzing cross-sectional data, it should be viewed as complementary to longitudinal studies rather than a replacement.

For each dataset, samples for lean and obese individuals were separately analyzed using BEEM-Static at the phylum level with the aim of first inferring the bacterial interaction within the community for each population (Méndez-Salazar et al., 2018), and then comparing the estimated carrying capacities and interaction coefficients between microbiota of lean and obese people.

Estimated carrying capacities for the lean and obese datasets were compared using pooled two-sample t-tests. Interaction coefficients that were identified as significant in both datasets were also compared in this way. The interaction strengths estimated by BEEM-Static represent the effect of one microbial phylum on another. A negative number indicates a competitive or inhibitory interaction, in which a rise in one phylum’s abundance suppresses the other. A positive score, on the other hand, indicates mutualistic or cooperative relationships, in which a rise in one phylum’s abundance benefits the other. Values close to zero, such as -0.1 or +0.1, indicate weak or negligible interactions, but values between -0.2 and -0.5, or +0.2 to +0.5, are considered moderate interactions. Values less than -0.5 or greater than +0.5 indicate significant interactions, with values approaching -1 or +1 indicating highly significant relationships. For instance, an interaction strength of -0.2 between Firmicutes and Bacteroidetes suggests moderate competition, whereas a value of -0.7 would signify a stronger competitive interaction. These thresholds provide a clearer framework for interpreting the significance and impact of interaction strengths within the gut microbiome. BEEM-Static also yields a coefficient of determination (R^2^) for each phylum that provides an indication of how well the model performed in predicted the sample relative abundances. The BEEM-Static package was downloaded from GitHub (Li et al., 2021), built in RStudio (version 1.4.1717), and compiled on a Windows 10 machine running R-4.0.2 (code available on https://github.com/enmelvan/Chapter4). To promote transparency and reproducibility, all datasets and analysis codes used in this study are publicly available on GitHub.

## 3 Results

### 3.1 Taxa analyzed

The total number of phyla retained for analysis ranged from 4 to 7 depending on the database, including *Actinobacteria*, *Bacteroidetes*, *Firmicutes*, *Lentisphaerae*, *Proteobacteria* and *Verrucomicrobia* (**Table 2**). These phyla were selected due to their known relevance in obesity-related gut microbiota research and their consistent presence across datasets.

**Table 2:** Sample - average relative abundances for the analyzed phyla across each database.

Dataset	Population	Actinobacteria	Bacteroidetes	Euryarchaeota	Firmicutes	Lentisphaerae	Proteobacteria	Verrucomi crobia	Unassigned
Turnbaugh	Obese	1.47%	27.68%	-	69.69%	-	1.1%	-	-
	Lean	1.04%	30.77%	-	67.4%	-	0.7%	-	-
HMP	Obese	0.45%	67.65%	-	31.15%	-	2.27%	-	-
	Lean	0.53%	67.67%	-	30.76%	-	2.2%	-	-
Goodrich	Obese	1.11%	29.76%	0.87%	62.94%	0.40%	2.19%	2.65%	1.21%
	Lean	0.94%	28.86%	1.23%	63.81%	0.41%	1.57%	3.15%	1.33%
Gordon	Obese	2.47%	30.94%	-	67.89%	-	-	-	-
	Lean	1.56%	38.52%	-	60.86%	-	-	-	-
AG	Obese	2.13%	45.61%	-	50.78%	-	1.21%	1.37%	-
	Lean	2.44%	44.54%	-	51.36%	-	1.27%	1.87%	-
Ross	Obese	0.20%	41.30%	-	57.23%	-	1.16%	-	0.89%
	Lean	0.11%	41.27%	-	57.17%	-	0.62%	-	0.71%

‘Unassigned’ refers to a small number of OTUs that could not be classified into a specific phylum in the Goodrich and Ross datasets.

### 3.2 Overall model fit

A total of 12 BEEM-Static analyses were performed, including one lean-population analysis and one obese-population analysis, for each of the 6 datasets. For each analysis, BEEM-Static yielded a set of estimates for carrying capacities and interaction coefficients that together can be used to predict the relative abundances of each phylum in each sample. Phylum-level coefficients of determination between the observed and predicted relative abundances consistently yielded R2 values greater than or equal to 0.55 (**Supplementary Table 1**), indicating that the fitted models provided robust descriptions of the data. The consistent R² values of ≥0.55 across datasets confirm the robustness of the BEEM-Static model in accurately predicting phylum-level relative abundances, underscoring its utility in analyzing gut microbiota dynamics in lean and obese individuals.

### 3.3 Carrying capacities

Estimated carrying capacities (**Supplementary Table 2**) differed significantly between the lean and obese datasets for most phyla in at least 4 of the 6 datasets (**Table 3**), with *Firmicutes* and *Bacteroidetes* generally having the highest values, as expected given their generally greater abundances. Interpretation of these differences are, however, challenging because the BEEM-Static algorithm estimated these parameters by setting the median total abundance estimate to an arbitrary default value of 1000, making these estimates sensitive to the average taxon richness of samples. The challenge in interpreting these differences arises from the arbitrary setting of the median total abundance estimate, which may affect the sensitivity of the carrying capacity estimates to variations in sample richness. We therefore will not discuss these results any further.

**Table 3:** Significantly different carrying capacity of phyla in lean and obese individuals.

Phyla	#	Lean	Obese
Firmicutes	*5*	*389.66*	*475.02*
Proteobacteria	*5*	*5.14*	*42.09*
Actinobacteria	*5*	*16.34*	*38.12*
Bacteroidetes	*4*	*229.38*	*265.92*

Only significant carrying capacities that appear in at least three databases are shown. The # column represents the number of datasets where the carrying capacity was significantly different, and the average BEEM coefficients for lean and obese populations were calculated.

### 3.4 Interactions summary

Among the 6 datasets, 57 significant interactions were identified for obese populations, 79% of which were negative, while only 37 significant interactions were identified for lean populations, 92% of which were negative (**Supplementary Table 3**; see https://github.com/enmelvan/Chapter4 for the complete set of significant interactions and statistical comparisons among coefficients). Two interactions were identified as significant only in the lean-data analyses, whereas 5 interactions were identified as only significant in the obese-data analyses. All of the interaction coefficients that were identified as significant, and of the same sign, in 3 or more databases were negative (**Table 4**), suggesting that the microbiota is predominantly structured by amensal and competitive interactions, at least at the phylum level. Negative effects of *Firmicutes* on the abundance *Bacteroidetes*, and *Bacteroidetes* on *Firmicutes*, were the most commonly observed interactions. The predominance of negative interactions in obese microbiotas suggests a competitive and possibly dysbiotic microbial environment, which could be a target for therapeutic interventions aimed at restoring a healthier balance.

**Table 4:** Interaction coefficients that were identified as significant, and of the same sign, in at least 3 of the 6 datasets.

Lean populations				Obese populations			
Origin	Target	#	Average Interaction Strength	Origin	Target	#	Average Interaction Strength
Firmicutes	Bacteroidetes	*6*	*-0.257*	Firmicutes	Bacteroidetes	*5*	*-0.264*
Bacteroidetes	Firmicutes	*5*	*-0.256*	Bacteroidetes	Firmicutes	*5*	*-0.41*
Actinobacteria	Firmicutes	*3*	*-1.401*	Actinobacteria	Firmicutes	*4*	*-0.134*
Actinobacteria	Bacteroidetes	*3*	*-0.723*	Proteobacteria	Firmicutes	*4*	*-1.151*
				Actinobacteria	Proteobacteria	*3*	*-0.23*
				Firmicutes	Proteobacteria	*3*	*-0.123*

The # column corresponds to the number of datasets used to calculate the averages.

**Figure 1:**
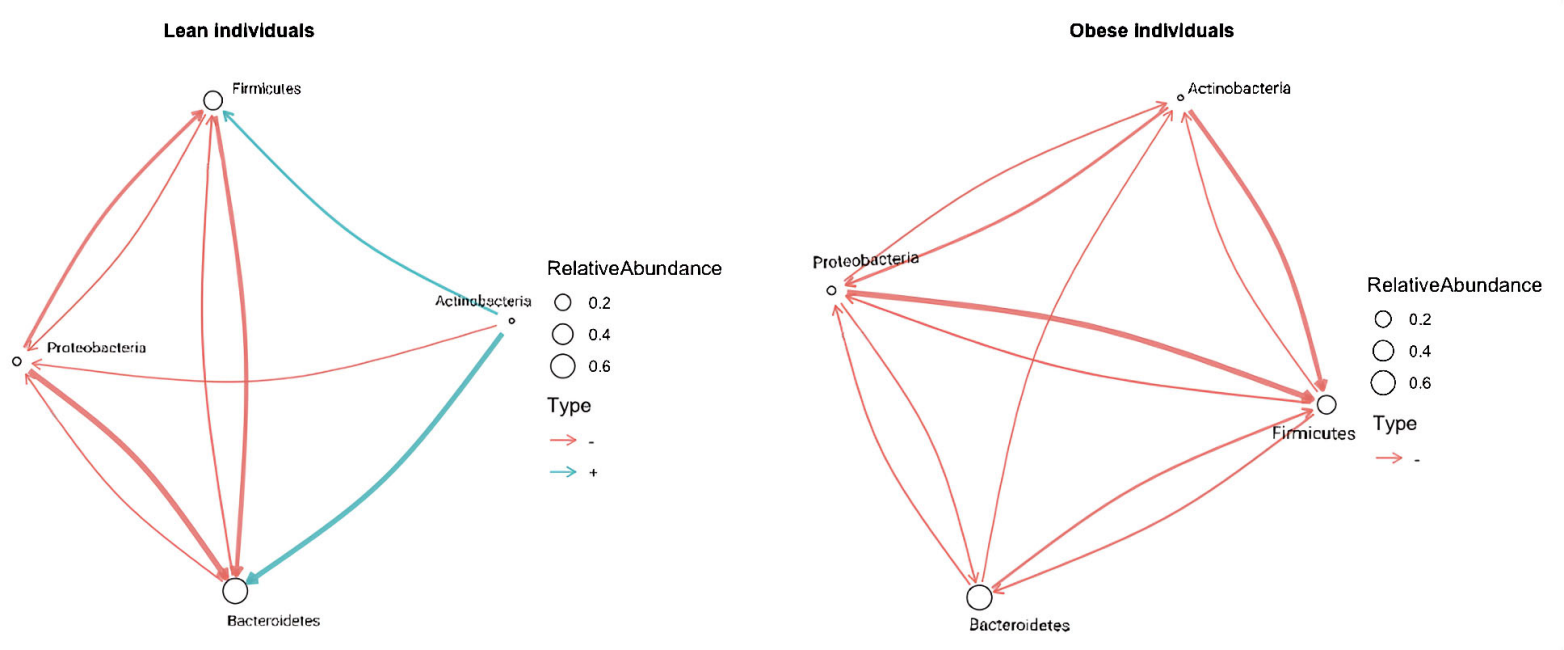
Microbial interaction network based on microbiome profiling of 217 lean and 185 obese fecal samples from lean patients in HMP data. A network graph indicating microbial interactions from GLVM model learned by BEEM-Static. Positive interactions are represented by blue, and negative relations are represented by red graph edges. Node sizes are proportional to the log-transformed mean relative abundance of the corresponding phylum, and edge thicknesses are proportional to the interaction strength. Taxonomic annotations at the phylum level are used to mark nodes.

The interaction coefficients identified as being significant by BEEM-Static can be represented as directed-network graphs (**Supplementary Figure 1**). These network graphs are crucial for visualizing and interpreting the complex interactions within the microbiota, providing a clear representation of the differences between lean and obese individuals. Using analyses of HMP data as an example (**Figure 1**), the BEEM-Static analyses indicate that increases in *Actinobacteria* abundance have a strong positive effect on *Bacteroidetes* abundance among lean people (denoted by a thick blue arrow in **Figure 1**), but a weak negative effect on *Bacteroidetes* abundance among obese people (denoted by a thin red arrow).

All notable differences in microbial interactions within lean and obese individuals can be found in **Table 5**, and all significant interactions can be found in https://github.com/enmelvan/Chapter4.

**Table 5:** Significantly distinct interactions in lean and obese individuals.

Origin	Target	#	Average interaction strength	Origin	Target	#	Average interaction strength
Firmicutes	Bacteroidetes	*3*	*-0.305*	Firmicutes	Bacteroidetes	*3*	*-0.368*
Bacteroidetes	Firmicutes	*3*	*-0.3167*	Firmicutes	Proteobacteria	*3*	*-0.123*
				Actinobacteria	Proteobacteria	*3*	*-0.23*
				Proteobacteria	Firmicutes	*3*	*-1.015*

Only interactions that appear in at least three databases are shown. The # column denotes the number of datasets in which the relationship appeared, and the interaction strength is the average of those interactions’ BEEM coefficients.

## 4 Discussion

### 4.1 Bacteroidetes and firmicutes

Five or more of the 6 datasets showed a significant negative effect of Firmicutes on Bacteroidetes, as well as a significant negative effect of Bacteroidetes on Firmicutes (**Table 4**), indicating that the two taxa are in competition with one another. Three of the 6 datasets yielded Bacteroidetes effects on Firmicutes that differed significantly between the lean and obese analyses. The average negative interaction strength from Bacteroidetes to Firmicutes in obese people was -0.4, compared to -0.26 in lean people.

In the lean population, there were just two significantly distinct interactions: the previously described Firmicutes to Bacteroidetes interaction and, not surprisingly, the Bacteroidetes to Firmicutes interaction. The observed competition between Bacteroidetes and Firmicutes suggests that dietary interventions targeting these phyla could be critical in managing obesity. It is important to note, however, that the predictive value of the *Firmicutes*-to-*Bacteroidetes* (F/B) ratio for obesity remains controversial. Several recent studies have reported inconsistent or non-significant associations between this ratio and obesity status. For instance, a 2023 study found no significant differences in the F/B ratio between obese and lean individuals, questioning its utility as a universal biomarker for obesity (Zhang et al., 2023). Similarly, a 2022 review highlighted that variations in diet, geography, and methodological approaches contribute to the heterogeneity of findings, and emphasized the need for more refined methods to assess microbiome composition and function (Cheng et al., 2022). Our findings support this perspective by shifting the focus from static abundance ratios to dynamic interaction patterns, which may offer more robust insights into dysbiosis and host-microbiome relationships. For example, high-fiber diets are known to favor Bacteroidetes, potentially offering a strategy to modulate this balance in favor of a healthy microbiome.

### 4.2 Actinobacteria

Another interaction that occurs in both lean and obese people is the negative interaction between *Actinobacteria* and *Firmicutes*, with the average strength of the interaction being much stronger in lean people than obese people (-1.4, versus -0.13, **Table 4**), which is consistent with the previous finding of higher *Actinobacteria* abundance in obese people’s gut microbiome (John and Mullin, 2016; Tseng and Wu, 2019; Castaner et al., 2018). *Actinobacteria* exhibited a negative interaction with *Bacteroidetes* in the lean population, with an interaction strength of -0.72; however, this interaction did not significantly differ between the lean and obese groups, which contradicts established findings in the literature (Turnbaugh et al., 2009; Clarke et al., 2012).

Given the strong interaction between *Actinobacteria* and other phyla, dietary components known to promote *Actinobacteria*, such as prebiotics, could be leveraged to influence these microbial dynamics positively. Our findings confirm the dual nature of *Actinobacteria’s* impact, wherein their abundance and interactions can shift from beneficial to detrimental based on the host’s metabolic state, emphasizing the importance of precision in microbiota-targeted interventions. Personalized dietary strategies based on microbiome profiling could mitigate the negative effects of *Actinobacteria* in obese individuals, such as promoting strains that enhance metabolic health, and simultaneously leveraging their beneficial potential in lean individuals through tailored prebiotic or probiotic formulations.

Our study has shown that the *Actinobacteria* and *Proteobacteria* interactions both with each other and with other phyla are both characteristic and significant within obese individuals, and both phyla appear as the network hub. This is also in accordance with the current literature, as both the *Proteobacteria* and *Actinobacteria* phyla are more prevalent in obese people (Castaner et al., 2018; Méndez-Salazar et al., 2018

The negative interaction from *Actinobacteria* to *Proteobacteria* was significantly different for the lean and obese populations, confirming a hypothesis of this meta-analysis. The interaction strength of *Proteobacteria* to *Actinobacteria* also differed significantly between the lean and obese populations.

The carrying capacity of *Actinobacteria* is also significantly higher in obese populations than lean populations in 5 datasets, and the strength of the coefficient was twice as large in the obese population, which is in line with the current research of *Actinobacteria* and obesity. The relative differences between groups are still relevant despite BEEM-Static’s arbitrary total abundance baseline (set at 1000). All carrying capacities are proportionately rescaled by the model, thus comparisons between groups within the same taxon have biological significance even when absolute values cannot be interpreted in isolation. For instance, persistently higher carrying capacities for *Firmicutes* and *Proteobacteria* in obese people across several datasets imply that these phyla might have a larger ecological niche in microbiomes linked to obesity, possibly as a result of changes in nutrient availability or microbial shifts brought on by inflammation. We acknowledge that differences in sample sizes across datasets may influence the estimation of carrying capacities. Although BEEM-Static is designed to be robust to varying sample numbers, smaller datasets may yield less stable estimates. Ideally, achieving reliable and generalizable conclusions at the level of individual datasets would require large, homogeneous cohorts—potentially exceeding 10,000 samples per study. However, due to practical constraints, our study relied on existing publicly available data. Therefore, while trends observed consistently across multiple datasets increase our confidence in the results, findings at the level of individual datasets should be interpreted with caution.

The contrasting roles of *Actinobacteria* underscore the need for personalized dietary interventions (Castaner et al., 2018). Tailoring nutrition based on individual microbiome profiles could mitigate the adverse effects associated with certain *Actinobacteria* in obese individuals while promoting beneficial strains in lean individuals

### 4.3 Proteobacteria

Interestingly, *Firmicutes* and *Actinobacteria* displayed negative interaction with the *Proteobacteria* phylum only in the obese data, consistent with recent evidence indicating that inflammatory host response promotes *Proteobacteria* growth (Shin et al., 2015). A negative interaction from *Proteobacteria* to *Firmicutes* was also observed only in the obese individuals.

The *Firmicutes* to *Proteobacteria* negative interaction and *Proteobacteria* to *Firmicutes* negative interaction are significantly different interactions among the obese populations, with the latter being much greater in absolute magnitude than the former (average of -1 compared to -0.12).

The prominence of *Proteobacteria* in obese individuals suggests that diets aimed at reducing inflammation, such as those rich in polyphenols, could help suppress this phylum’s overgrowth and improve gut health.

### 4.4 Single database significant differences

Some significant interactions found in only one database are also worthy of note. In the HMP database, *Bacteroidetes* had a positive interaction with *Actinobacteria* in lean individuals, while in obese individuals, this interaction was negative. In the American Gut database, *Actinobacteria* had a negative interaction with *Firmicutes* in lean and obese individuals. In the Goodrich database, *Verrucomicrobia* and *Lentisphaerae* were also identified as having significant interactions that ranged from negative (obese population) to positive (lean population).

These findings underscore the importance of considering geographic and dietary differences when developing personalized nutrition strategies. For instance, dietary interventions effective in Western populations may need to be adjusted for Eastern diets to achieve similar microbiome modulation.

Also, only one interaction with *Lentisphaerae* was identified in the lean population, an unassigned bacterium, and this taxon had a positive effect on it. In obese individuals, a total of 10 different interactions were identified with *Verrucomicrobia* and *Lentisphaerae*. There is still not much research on these two phyla, except one on Chinese children and adolescents with obesity, which found that *Verrucomicrobia* and *Lentisphaerae* were both significantly lower in obese group than those in the control group (Hou et al., 2017). It should be noted that all the databases used in this meta-analysis involved people from Western countries, which have a very different diet than the Chinese population. Assessing potential differences between eastern and western countries is a topic worthy of further investigation due to geographically dietary differences.

Most of the interactions identified were negative (96%) and all the extracted interactions can be found in https://github.com/enmelvan/Chapter4. While most ecological interaction networks consist of positive and negative interactions in approximately the same ratio, results here indicate that interactions in the gut microbiome were mostly negative. While most ecological interaction networks include a balance of positive and negative relationships, our results indicate that gut microbiome interactions, particularly in obese individuals, are predominantly negative. This may be partially explained by the fact that the number of gut bacteria is relatively constrained, such that an increase in one taxon’s abundance often influence a decrease in another’s. Beyond this, such patterns may reflect a disrupted microbial ecosystem, marked by reduced diversity and intensified competition. In obese individuals, dominant taxa can suppress others more aggressively, leading to competitive exclusion and instability. Diet likely plays a role as well: low-fiber, high-fat diets, which are more common in obesity, reduce ecological niches and shared resources, potentially amplifying these antagonistic interactions (Shin et al., 2015; Castaner et al., 2018).

As noted in the Introduction, the largest meta-analysis of human microbiome links to obesity was conducted in 2014 by Walters et al, who identified few consistent relationships between obesity and species composition. In particular, the *Firmicutes: Bacteroidetes* ratio yielded no consistent patterns. This is why it is critical to investigate the dynamics of microbial communities. Quantifying interactions among taxa would help us better understand the ecology of the gut microbiome and better predict how perturbations will affect it. Removing a taxon that engages in mutualistic interactions, for example, may reduce the number of other taxa that rely on it for survival. Given their importance in understanding community ecology, there is a significant amount of interest in developing techniques to infer taxa interactions from metagenomic data.

One of the most serious restrictions of the presented meta-analysis is that network modeling could not be done on a merged dataset composed of all six different databases. Initially, we attempted to merge these datasets and applied XGBoost to identify patterns, but this approach did not yield meaningful results, as no clear pattern related to BMI was observed (Chen and Guestrin, 2016) (see **Supplementary Data** for XGBoost results). Consequently, we analyzed each database individually because cluster analyses performed on the combined dataset revealed no pattern related to BMI (see **Supplementary Data**; **Table 2**). Instead, data clustered strongly by study, with no consistent BMI pattern after controlling for study, consistent with prior work (Walters et al., 2014). These findings suggest that per-study effects are much larger than biological effects differentiating lean from obese people. Differences between studies are typically caused by technical and/or clinical factors. This finding emphasizes the need to control for factors inducing heterogeneity among studies because a taxonomic signature will need to be consistent across populations in order to be useful in a clinical setting. Even though inferring interactions at this taxonomic level is challenging due to the significant levels of heterogeneity among phyla in terms of metabolic and other features of microorganisms, the findings presented here demonstrate its utility. While BEEM-Static was chosen for its ability to infer ecological interactions from cross-sectional data, we acknowledge that alternative methods such as SPIEC-EASI, CoNet, and machine learning classifiers (e.g., random forest) could offer complementary insights. These models differ in their assumptions, with some focusing on co-occurrence networks and others on predictive power. In contrast, BEEM-Static provides an ecological modeling framework grounded in generalized Lotka-Volterra dynamics, enabling estimation of both interaction strengths and carrying capacities. Future research should consider benchmarking BEEM-Static against these approaches on harmonized datasets to assess performance and interpretability.

In our research, we did data pruning in order to keep only taxa that appeared in at least one-third of samples, it is important to mention that the low-abundant species also deserve attention. The precise investigation of low-abundant species is a difficult task since low-abundance bacterial species’ exact quantities are notoriously difficult to determine and use to draw conclusions about underlying mutualistic relationships (Jousset et al., 2017; dos Santos, 2012).

## 5 Conclusion

The industry is moving toward personalized nutrition based on gut bacteria, a new and rapidly evolving field in intestinal microbiota research. Recent medical advancements have demonstrated that human reactions to dietary stimuli are influenced by specific and quantifiable host and microbiome characteristics, rather than a one-size-fits-all diet (Kumar et al., 2019; Li et al., 2019; Malcolm, 1966; Jones et al., 1997). This study underscores the importance of understanding microbial interactions to tailor dietary interventions that can effectively modulate the gut microbiota toward a healthier composition, helping to meet nutritional needs and combat malnutrition and obesity.

Modulating the gut microbiota to better utilize available food and improve nutritional status—such as extracting more energy, minerals, or vitamins—is essential to overcoming today’s challenge of obesity. Through 16S rRNA gene analysis of the gut microbiota, we can gain insights into the diversity and abundance of microbiome components, as well as extract valuable information about pro-inflammatory bacteria, butyric acid-producing bacteria, and bacteria associated with protein and fat consumption.

Importantly, this approach allows for the extraction of meaningful insights from a single time point, making it a practical and scalable tool for personalized nutrition and gut microbiota modulation. By using this as a foundation, and by employing models like the BEEM model and the interactions identified in this study, we can make targeted food recommendations that shift the microbiota towards that of a lean population.

We name this dietary gut-brain axis intervention approach ‘*Optibiomics*’, representing a novel, AI-driven strategy to optimize gut health and combat obesity. Future research should explore the application of *Optibiomics* in diverse populations and its potential to address other microbiome-related conditions. While *Optibiomics* represents a promising approach based on our findings, we acknowledge that it currently lacks experimental or clinical validation. Future studies are essential to substantiate these claims through rigorous testing of personalized dietary interventions.

## Data Availability

The original contributions presented in the study are included in the article/**Supplementary Material**. Further inquiries can be directed to the corresponding author.
