# Supplementary material for: Predicting gut microbiota dynamics in obese individuals from cross-sectional data
Source: Front Cell Infect Microbiol. 2025 Jun 10;15:1485791. doi: 10.3389/fcimb.2025.1485791 (PMC12185450; doi:10.3389/fcimb.2025.1485791)
Supplement: Supplementary file 1 [file DataSheet1.pdf]

frontiers

# *Supplementary Material*

## 1 Supplementary Data

### 1.1 Datasets

This study analyzed six publicly available datasets that include gut microbiome profiles from lean, overweight, and obese individuals. Each dataset was processed to extract relevant demographic and phenotypic information, summarized below.

#### 1.1.1 American Gut (AG)

The American Gut dataset includes 3,202 samples, distributed across two phenotypic groups:

- **H (Healthy):** 1,906 samples (1,013 female, 893 male), mean age 42 years (range: 0–93).
- **OB (Obese):** 1,296 samples (697 female, 599 male), mean age 49 years (range: 0–93).

#### 1.1.2 Turnbaugh

The Turnbaugh dataset consists of 142 samples, categorized into two phenotypic groups: 35 lean and 107 obese individuals. No information on age or sex is available for this dataset.

#### 1.1.3 Ross

The Ross dataset includes 63 samples, distributed across two phenotypic groups:

- **H (Healthy):** 6 samples (3 male, 3 female), mean age 61.8 years (range: 55–71).
- **OB (Obese):** 57 samples (45 female, 12 male), mean age 56.6 years (range: 33–81).

#### 1.1.4 Human Microbiome Project (HMP)

The HMP dataset includes 6,203 samples, with the following phenotypic distribution:

- **H (Healthy):** 2,646 samples (1,452 female, 1,194 male), mean age 25.9 years (range: 18–40).
- **OB (Obese):** 3,557 samples (2,203 male, 1,354 female), mean age 28.0 years (range: 18–40).

#### 1.1.5 Gordon

The Gordon dataset contains 281 samples, categorized into two phenotypic groups:

- **H (Healthy):** 61 samples.
- **OB (Obese):** 220 samples.

No detailed demographic information such as age or sex is available for this dataset.

### 1.1.6 Goodrich

The Goodrich dataset includes 1,017 samples distributed as follows:

- **H (Healthy):** 538 samples, mean age 60.6 years (range: 23–86).
- **OB (Obese):** 479 samples, mean age 61.3 years (range: 32–79).

## 1.2 Xgboost for data clustering

The XGBoost (eXtreme Gradient Boosting) package is a popular machine learning decision-tree-based algorithm that uses a gradient boosting method [36,37]. Gradient boosting is a form of supervised learning algorithm that incorporates the estimates of a series of simpler, weaker models in an attempt to correctly predict a target variable. The application of the XGBoost to all six databases completely failed to separate the obese from lean samples based on the OTU table in five out of six utilized databases, with prediction accuracy no better than random  and p-values between 0.32 and 1. (See the Supplement Table 4 for complete XGBoost results.) Thus, our findings align with the conclusions of the 2014 meta-analysis (Knight et al.,2014). Only in the HMP database dataset the accuracy of the clustering separation is 0.68 with the p-value 0.00015.

# 2 Supplementary Figures and Tables

**Table S1**. Coefficient of determination for each phylum in each data dataset.

| Dataset | Population | Actinobacteria | Bacteroidetes | Euryarchaeota | Firmicutes | Lentisphaerae | Proteobacteria | Verrucomicrobia | Unassigned |
|---|---|---|---|---|---|---|---|---|---|
| AG | Obese | 0.998 | 0.998 | - | 0.997 | - | 0.928 | - | - |
| | Lean | 0.76 | 0.87 | - | 0.85 | - | 0.55 | - | - |
| Goodrich | Obese | 0.998 | 0.999 | - | 0.996 | 0.984 | 0.997 | 0.998 | 0.997 |
| | Lean | 0.997 | 0.998 | - | 0.993 | 0.983 | 0.999 | 0.998 | 0.996 |
| Gordon | Obese | 0.992 | 1 | - | 0.999 | - | - | - | - |
| | Lean | 0.991 | 1 | - | 0.999 | - | - | - | - |
| HMP | Obese | 0.55 | 0.999 | - | 1 | - | 1 | - | - |
| | Lean | 0.82 | 0.999 | - | 1 | - | 0.999 | - | - |

| | | | | | | | | | |
|---|---|---|---|---|---|---|---|---|---|
| Ross | Obese | 0.8 | 0.63 | - | 0.62 | - | 0.76 | - | 0.625 |
| | Lean | 0.941 | 0.965 | - | 0.946 | - | 0.981 | - | 0.932 |
| Turnbaugh | Obese | 0.989 | 0.91 | - | 0.57 | - | 0.91 | - | - |
| | Lean | 0.998 | 0.999 | - | 0.997 | - | 0.983 | - | - |

**Table S2**. Estimates carrying capacities for each phylum and dataset. The absolute values are arbitrarily set so that the median total abundance of a sample is equal to 1000, making these estimates sensitive to the average sample richness.

| Dataset | Population | Actinobacteria | Bacteroidetes | Euryarchaeota | Firmicutes | Lentisphaerae | Proteobacteria | Verrucomicrobia | Unassigned |
|---|---|---|---|---|---|---|---|---|---|
| AG | Obese | 0.6795 | 503.8054 | - | 423.1411 | - | 11.7457 | - | - |
| | Lean | 6.2873 | 245.7523 | - | 308.3550 | - | 1.7438 | - | - |
| Goodrich | Obese | 0.2212 | 15.6332 | - | 636.0885 | 4.3707 | 0.2012 | 3.9161 | 8.7182 |
| | Lean | 0.1833 | 81.4999 | - | 177.7509 | 0.4480 | 0.1176 | 0.5201 | 1.3959 |
| Gordon | Obese | 67.2244 | 151.0946 | - | 700.5462 | - | - | - | - |
| | Lean | 67.9563 | 216.0668 | - | 601.9640 | - | - | - | - |
| HMP | Obese | 120.5937 | 393.1480 | - | 296.6367 | - | 192.2044 | - | - |
| | Lean | 10.0786 | 374.2114 | - | 272.2653 | - | 18.3433 | - | - |
| Ross | Obese | 0.82269 | 234.0344 | - | 254.596 | - | 12.0446 | - | 3.7425 |
| | Lean | 1.5771 | 189.2567 | - | 324.4487 | - | 1.2801 | - | 1.4464 |
| Turnbaugh | Obese | 1.0076 | 142.2787 | - | 318.6819 | - | 2.5780 | - | - |
| | Lean | 2.6627 | 353.0776 | - | 588.0053 | - | 4.2212 | - | - |

**Table S3**. Tallies of positive and negative interactions among the 12 analyses.

| **Lean Population** | | | | **Obese Population** | | | |
|---|---|---|---|---|---|---|---|
| **Origin** | **Target** | **Direction** | **#** | **Origin** | **Target** | **Direction** | **#** |
| Firmicutes | Bacteroidetes | neg | 3 | Firmicutes | Proteobacteria | neg | 3 |
| Bacteroidetes | Firmicutes | neg | 3 | Actinobacteria | Proteobacteria | neg | 3 |
| Actinobacteria | Proteobacteria | neg | 2 | Bacteroidetes | Firmicutes | neg | 3 |
| Firmicutes | Actinobacteria | | | Firmicutes | Bacteroidetes | neg | 3 |

| | | | | | | | |
|---|---|---|---|---|---|---|---|
| Firmicutes | Proteobacteria | neg | 2 | Proteobacteria | Firmicutes | neg | 3 |
| Actinobacteria | Bacteroidetes | neg | 2 | Firmicutes | Actinobacteria | neg | 2 |
| Bacteroidetes | Actinobacteria | neg | 2 | Bacteroidetes | Actinobacteria | neg | 2 |
| Proteobacteria | Actinobacteria | neg | 2 | Actinobacteria | Bacteroidetes | neg | 2 |
| Bacteroidetes | Proteobacteria | neg | 2 | Actinobacteria | Firmicutes | neg | 2 |
| Proteobacteria | Bacteroidetes | pos | 1 | Actinobacteria | Firmicutes | pos | 2 |
| Proteobacteria | Firmicutes | neg | 1 | Proteobacteria | Actinobacteria | neg | 2 |
| Actinobacteria | Firmicutes | neg | 1 | Bacteroidetes | Proteobacteria | neg | 1 |
| Bacteroidetes | Proteobacteria | pos | 1 | Proteobacteria | Bacteroidetes | neg | 1 |
| | | | 1 | Firmicutes | Bacteroidetes | pos | 1 |

**Table S4.** Results of the XGBoost clustering.

| | **Accuracy** | **95% CI** | **P-Value** | **Sensitivity** | **Specificity** | **Balanced Accuracy** |
|---|---|---|---|---|---|---|
| AG | 0.5134 | (0.4638, 0.5629) | 1.00000 | 0.5741 | 0.3957 | 0.4849 |
| Turnbaugh | 0.5091 | (0.4829, 0.5352) | 0.3269 | 0.5097 | 0.5084 | 0.5090 |
| HMP | 0.6811 | (0.6252, 0.7334) | 0.0001539 | 0.6378 | 0.7126 | 0.6752 |
| Goodrich | 0.4687 | (0.4321, 0.5055) | 1.00000 | 0.5152 | 0.5152 | 0.5540 |
| Gordons | 0.7095 | (0.6431, 0.7699) | 0.9999969 | 0.51613 | 0.82123 | 0.66868 |
| Ross | 0.4255 | (0.2826, 0.5782) | 1.0000 | 0.25000 | 0.48571 | 0.36786 |

## 2.1 Supplementary Figures

(A) American Gut

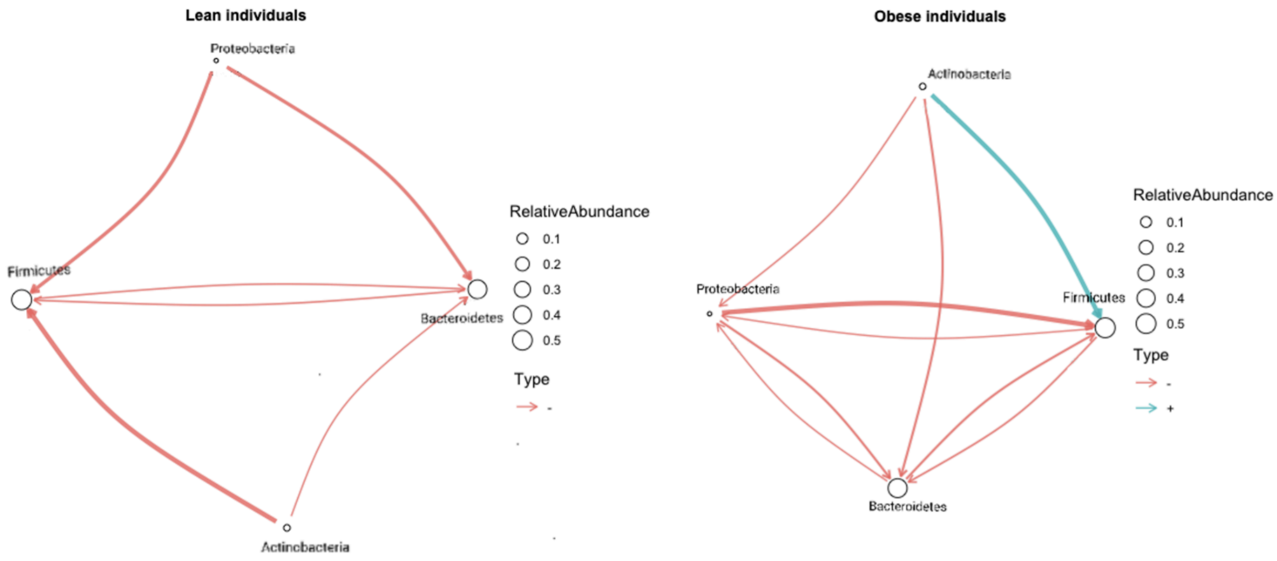

(B) Goodrich

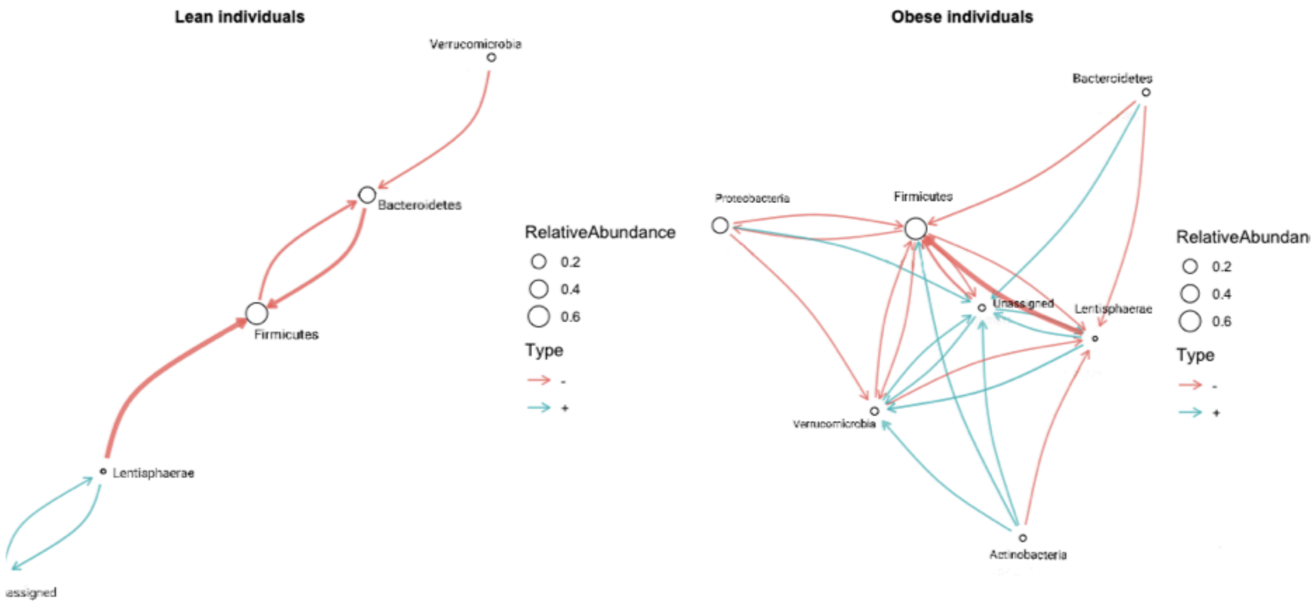


(C) Gordon

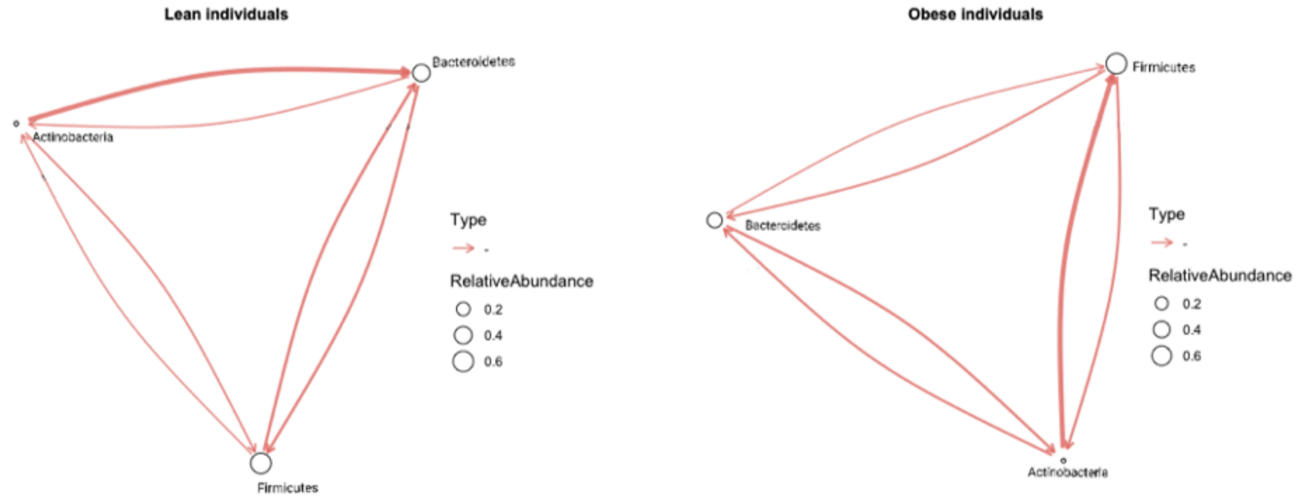

(D) Ross

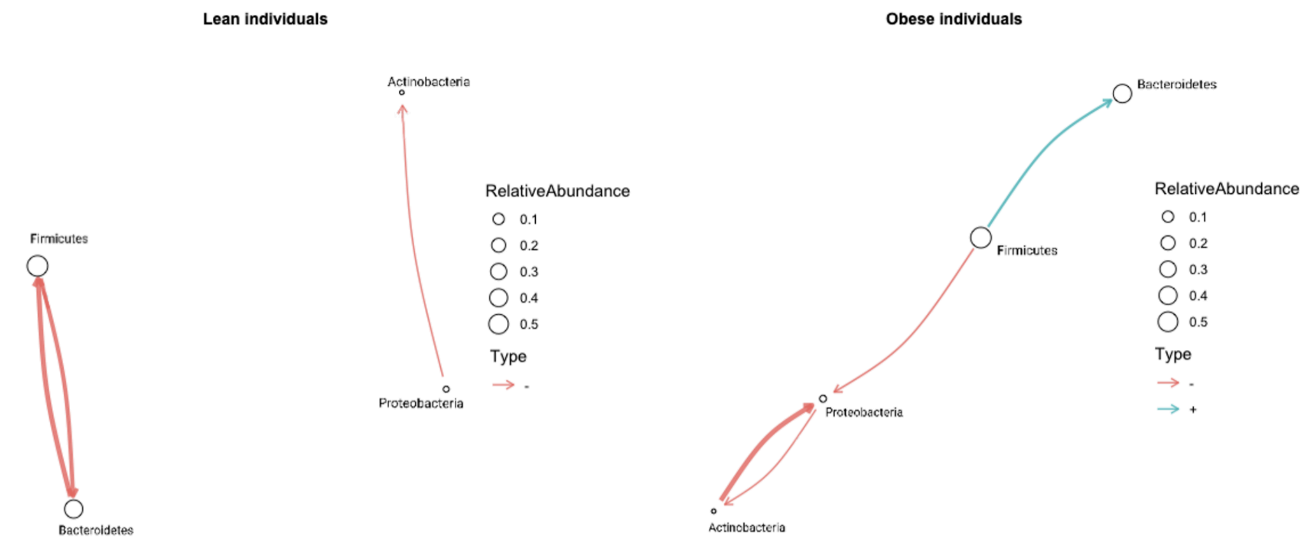


(E) Turnbaugh

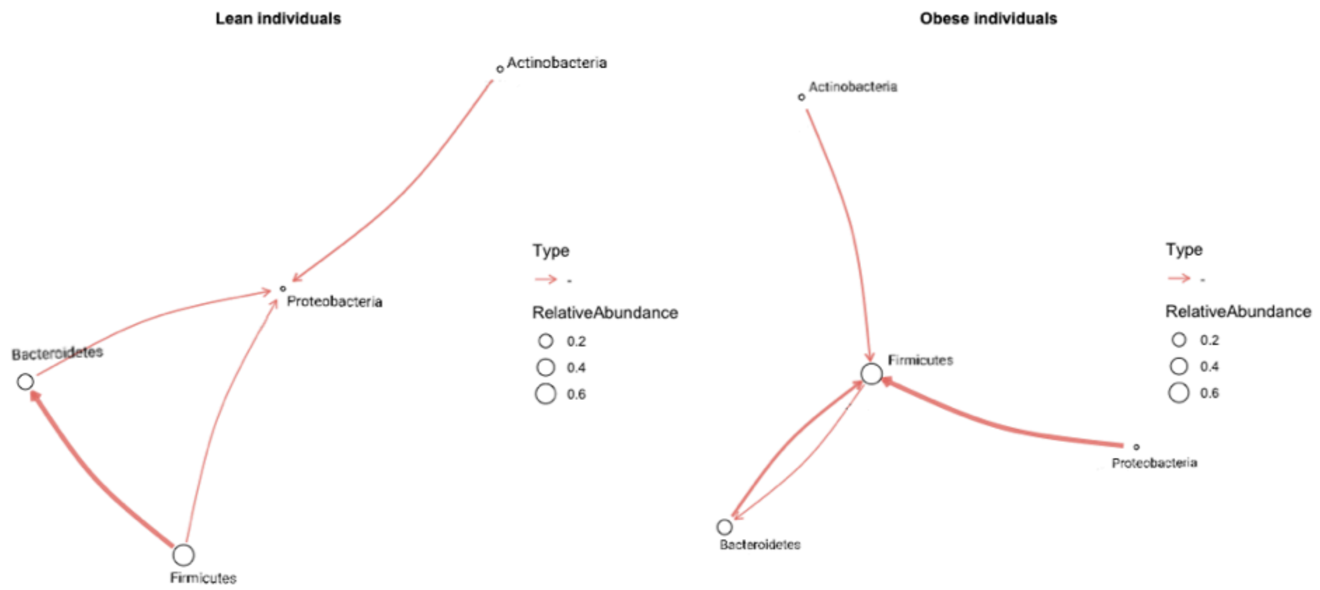

Supplementary Material

**Supplementary Figure 1.** Significant generalized Lotka Volterra model interaction coefficients for other 5 databases listed in Table S1 ((A) American gut; (B) Goodrich; (C) Gordon; (D) Ross; (E) Turnbaugh), as identified by BEEM-Static, are depicted as directed network graphs with phyla as nodes. Positive interactions are represented in blue, and negative interactions are represented by red graph edges. Node sizes are proportional to the log-transformed mean relative abundance of the corresponding phylum, and edge thicknesses are proportional to the interaction strength.
